# Quality of neonatal resuscitation in Ethiopia: implications for the survival of neonates

**DOI:** 10.1186/s12887-020-02029-5

**Published:** 2020-03-19

**Authors:** Haftom Gebrehiwot Weldearegay, Mulugeta Woldu Abrha, Esayas Haregot Hilawe, Brhane Ayele Gebrekidan, Araya Abrha Medhanyie

**Affiliations:** 1 College of Health Sciences, Mekelle University, Mekelle, Ethiopia; 2 Tigray Health Research Institute, Mekelle, Ethiopia

**Keywords:** Neonatal resuscitation, Quality of care, Emergency obstetrics and newborn care, Birth asphyxia and Ethiopia

## Abstract

**Background:**

Birth asphyxia accounts for one-quarter newborn deaths. Providing quality care service of neonatal resuscitation reduces neonatal mortality. However, challenges to providing quality neonatal resuscitation are not well investigated in Ethiopia. Hence, this study is conducted to assess the quality provision of neonatal resuscitation in Ethiopia.

**Method:**

We used data from the Ethiopian 2016 Emergency Obstetric Newborn Care survey, conducted in 3804 health facilities providing maternal and newborn health services. We described the quality of neonatal resuscitation services according to the structure, process and outcome triad of quality dimension. Data from registers and birth records for the last 12 months prior to the survey were extracted. In each facility, the three last eligible charts of resuscitated neonates were reviewed and the highest frequency of chart of resuscitated baby was considered to the analysis. Thus, a total of 555 charts were assessed. Logistic regression model was used to assess the relationship between the neonatal resuscitation processes, provider, facility and newborn characteristics with neonatal outcome at the time of discharge.

**Results:**

The finding suggested that, around two-third, 364(65.6%) of the asphyxiated babies resuscitated by bag and mask type of neonatal resuscitation. Of the babies who had got neonatal resuscitation 463 (83.4%) survived. Resuscitated neonates with a gestational age of greater than 37 weeks and above (Adjusted Odds Ratio (AOR) =1.82; 95% Confidence Interval (CI) (1.09–3.04)), availability of priority equipment in health facilities for neonatal resuscitation (AOR = 1.24, 95% CI (1.09, 1.54)) and women who had 12 h and less duration of labor (AOR = 1.76; 95% CI (1.23, 3.13)) were the independent factors of survival of the neonate.

**Conclusion:**

Only half of the health facilities were ready for neonatal resuscitation (NR) in terms of priority equipment’s. However, eight out of ten babies survived after NR in Ethiopia. Gestational age, priority equipment for NR and duration of labor were determinants of survival of resuscitated neonates in Ethiopia. Therefore, the availability of priority equipment and attentive care and follow-up for premature neonates and those face prolonged labor need to be improved in Ethiopia.

## Background

Birth asphyxia is defined by the World Health Organization (WHO) as “the failure to initiate and sustain breathing at birth” and accounts for one-fourth of neonatal mortality [1]. Each year an estimated 10 million babies require assistance to start breathing. Five to 10 % of babies born in facilities need some degree of resuscitation, like tactile stimulation or airway clearing or positioning. Approximately 3–6% requires basic neonatal resuscitation, consisting of certain simple initial steps and assisted ventilation [2]. There is an urgent need for neonatal resuscitation in low-resource settings, where access to intrapartum obstetric care is poor and the incidence, mortality, and burden of long term impairment from intra-partum-related events is very high. Delays in initiating resuscitation may exacerbate hypoxia, and leads to neonatal morbidity and mortality [3, 4]. Evidence showed that risk of death increases by 16% for every 30 s delay in initiating ventilation up to 6 min and every 6% for every minute of delay [5]. Therefore, it is clear that the first minute after birth is critical to reduce neonatal mortality. Furthermore, successful neonatal resuscitation by well – trained health care providers has a potential to prevent perinatal mortality due to intrapartum related asphyxia [6]. Newborns with birth asphyxia can suffer from short- to long-term neurological complications [7]. Therefore, urgent referral of complicated birth asphyxia cases to higher well specialized facility is mandatory. As per the updated recommendations of American Heart Association and American Academy of Pediatrics -international liaison committee on resuscitation; neonatal resuscitation program requires at least one trained person to be present during delivery [8]. This requires that the healthcare personnel involved need to be abreast with the latest recommendations and should follow them in their clinical practice.

According to the EDHS 2016, the neonatal mortality rate in Ethiopia was 29 per 1000 live births [9]. In low-income countries ineffective resuscitation practices are linked insistently to high neonatal deaths from birth asphyxia in the first24 hours [10]. Besides, poor record–keeping and inconsistent quality of care is a major impediment to efforts aimed at improving the health of neonates. It is suspected that while coverage of institutional delivery services have been increased, the quality of care provided is substandard [9–12]. Therefore, this 2016 national Emergency Obstetrics and Newborn Care survey [13] provides a unique opportunity to address the information gap of the capacity of quality of neonatal resuscitation to treat and manage asphyxiated babies. In this analysis, we aimed to explore the factors associated with the neonate’s survival after undergoing neonatal resuscitation.

## Methods

### Data source

This was a secondary analysis of the data collected by the 2016 Ethiopian Emergency Obstetrics and Newborn care (EmONC) survey [13]. The EmONC assessment was a national cross-sectional survey of all public hospitals, health centers and private facilities (higher clinics and above) that provided maternal and newborn health services and reported attending births in the past 12 months. The EmONC assessment did not include health posts or medium and small private clinics because these facilities are not expected to attend deliveries. Of the eligible 4385 facilities in all nine regions and two city administrations in Ethiopia, 3804 facilities were assessed (including 293 hospitals, 3459 health centers and 52 clinics). A total of 11 facilities were not accessible due to political unrest or staff refusal. The survey used 13 questionnaires including 12 health facility assessment modules and one health system assessment module. These were adapted from the Averting Maternal Death and Disability program. A module on newborn complications was designed to collect information on resuscitated babies. Data from registers and birth records for the last 12 months prior to the survey were also extracted. In each facility, the three last eligible charts of resuscitated neonates were reviewed and the highest frequency of chart of resuscitated baby were extracted and enrolled to the analysis. Thus, a total of 555 charts were assessed with regard to the process of NR, provider, facility and resuscitated asphyxiated babies characteristics and neonate’s outcome [13, 14].

### Measurements

Our primary outcome of interest was the newborn’s outcome status after resuscitation had been done to the asphyxiated baby. Neonatal resuscitation refers to all the resuscitation steps: Stimulation, Bag and mask, stimulation and bag and mask, Intubation and asphyxiated babies were when the newborn has at least one of the following signs, not breathing, gasping, < 30 breath per minute or < 7 Apgar score [15]. It had a binary outcome: survived or dead. Explanatory variables influencing newborn survival after undergoing NR were measured through the Donabedian domains of structure and process quality indicators from the different literatures in line with our secondary data. The process metrics measured using all steps/ type of NR. Furthermore, a percentage of composite mean indexes created for NR service readiness in accordance with the WHO service readiness assessment approach [14]. Facility types were based on definitions by the Federal Ministry of Health. Hospitals generally have operating theaters while health centers and clinics do not [13].

### Data analysis

We used logistic regression to assess the association between neonatal outcome and type of neonatal resuscitation, health facility and provider characteristics. Because only one resuscitated babies chart was enrolled in each facility, this unit of analysis was the facility, and the outcome of interest was whether or not the neonate had survived after NR was performed. The explanatory variables for neonate’s outcome after NR were characteristics of facilities with asphyxiated baby and characteristics of providers who performed NR. A Bi-variable logistic regression analysis was conducted and those independent variables with *p* value of ≤0.25 were considered for inclusion in the multivariable logistic regression model with the forward likelihood ratio method. Finally, variables with *p* < 0.05 in the multivariable analysis were considered to declare statistically significant associations between covariates and neonate’s survival after NR. All analyzes were performed using SPSS version 21™ software.

## Results

### Sample

The EmONC national survey included a total of 3804 health facilities that offered delivery services from both government and private health facilities (referral, general, primary hospitals), health centers and clinics). Of the 3804 facilities surveyed by EmONC, charts of resuscitated babies were reviewed in only 555 facilities (14.6%).

### Structural quality

#### NR service-specific readiness

The most widely available essential medicine and commodities in the maternity area prepared for NR were neonatal Ambu-bag or ventilator [96.9%], mucus extractor (94.6%), and Vitamin K (94.2%). Meanwhile, chlorohexidine (4% gel for cord cleansing) was only available in 15.8% and towel about 38% of the health facilities. In relation to the components of priority of equipment’s for NR was fetoscope [95.5%], syringes of 0.5 or 1 ml volume [87.4%], and radiant warmer only available in 57.7% of the health facilities.

The overall availability of infrastructural readiness was 64.2%, availability of essential medicine and commodities 69.4%, priority equipment’s 51.5% and national helping baby breath guidelines 82.5% of the health facilities in Ethiopia (Table 1)**.**

**Table 1:** Structural readiness of health facilities for NR services in Ethiopia, EmONC, 2016, (*N* = 555)

Characteristics	Frequency	Percentage
Availability of essential medicine and commodities for NR		
Mucus extractor		
Yes	525	94.6
No	30	5.4
Neonatal size ambu (ventilator) bag		
Yes	538	96.9
No	17	3.1
Infant face masks (sizes 0,)		
Yes	450	81.1
No	105	18.9
Infant face masks (sizes 1)		
Yes	448	80.7
No	107	19.3
Towels/blanket or cloth for newborn		
Yes	212	38.2
No	343	61.8
Vitamin K (for newborn)		
Yes	520	94.2
No	32	5.8
Chlorehexidine (4% gel for cord cleansing)		
Yes	87	15.8
No	465	84.2
New born resuscitation table		
Yes	494	89.0
No	61	11.0
Availability of essential medicine and commodities (mean ± SD) of 9 items		6.9 ± 1.3
Priority equipment’s for NR:		
Syringes (0.5, 1 ml)		
Yes	485	87.4
No	70	12.6
Radiant warmer		
Yes	320	57.7
No	235	42.3
Fetal stethoscope		
Yes	530	95.5
No	25	4.5
Watch/clock		
Yes	306	55.1
No	249	44.9
Mucus trap for suction / suction apparatus		
Yes	299	53.9
No	256	46.1
Availability of priority equipment’s (mean ± SD) of 5 items		3.5 ± 1.1
Infrastructure of 10 components^a^ (mean % (SD)		7.9 ± 1.5
Availability of neonatal resuscitation guideline		
Yes	458	82.5
No	97	17.5
Number of deliveries in past 12 months (mean ± SD)		684.5 ± 826

^a^Electricity functional, Water, generator, Telephone, Radio, Ventilation, Toilet, Light source, fan/ air conditioning, and waiting area

#### Health care providers’ background characteristics

A total of 555 health care providers (HCPs) with a mean age of 26.1 years (SD ± 5.9) participated in the study. Almost more than half of HCPs (*n* = 334; 60.2%) were aged less than 25 years. Midwives account the majority of professional cadre (*n* = 504; 90.8%) to provide newborn resuscitation. Four out of ten (*n* = 243; 43.8%) participants indicated having less than 2 years’ experience. Over three fourth of the HCPs, 471(84.9%) reported having received the NR training within the past two years prior to this study (Table 2)**.**

**Table 2:** Characteristics of providers who performed NR, EmONC, 2016 (*N* = 555)

Characteristics	Frequency	Percentage
Professional Cadre		
MD/Health officer	16	2.9
Midwife	504	90.8
Nurse	35	6.3
Work experience in years		
< 2	243	43.8
2–5	236	42.5
> 5	76	13.7
Age in completed years		
< 25	334	60.2
25–40	206	37.1
> 40	15	2.7
Sex of care provider		
Female	341	61.4
Male	214	38.6
Residence of care provider		
Urban	283	51.0
Rural	272	49.0
Providers trained NR		
Yes	471	84.9
No	84	15.1

#### Health facility characteristics

Four of the ten health facilities reported having a separate newborn corner at the time of the survey and majority (*n* = 472; 85%) of health facilities reported that they didn’t have separate Neonatal Intensive Care Unit (NICU). Less than half (*n* = 249; 49.9%) of the facilities reported that have frequent staff rotation for newborn care. High proportion of health centers (*n* = 408; 80.7%) was included in this study. Concerning operating agency, almost all health facilities (*n* = 534; 96.2%) were operated by government (Table 3)**.**

**Table 3:** Characteristics of health facilities, EmONC survey, Ethiopia, 2016 (*N* = 555)

Characteristics	Frequency	Percentage
Facility has a separate newborn corner		
Yes	232	41.8
No	323	58.2
Facility has separate NICU		
Yes	83	15.0
No	472	85.0
Facility has staff rotation policy for newborn care services		
Yes	249	44.9
No	306	55.1
Facility type		
Hospitals	107	19.3
Health centers	448	80.7
Operating agency		
Government	534	96.2
Private	21	3.8

### Neonatal resuscitation process quality and outcomes

Around two third, (*n* = 364; 65.6%) of the asphyxiated babies were resuscitated using bag and mask, whereas, only 9(1.6%) was done by stimulation.

Overall, regarding outcome of the neonates after resuscitation, majority of the neonates (*n* = 463, 83.4%) were survived (Table 4)**.**

**Table 4:** NR process quality and outcomes in Ethiopia, EmONC survey 2016 (*N* = 555)

Characteristics	Frequency	Percentage
Neonatal resuscitation steps		
Stimulation	9	1.6
Bag and mask	364	65.6
Both stimulation and bag and mask	171	30.8
Intubation	11	2.0
Outcome of neonatal resuscitation for the asphyxiated babies at time of discharge		
Survived	463	83.4
Not survived	92	16.6

### Factors associated with outcome of neonatal resuscitation

Table 5 examined the relationship between the explanatory variables of NR processes, newborn characteristics, health providers and health facility characteristics on neonatal survival at the time of discharge. Thus, in bi-variable analyses mode of delivery, birth weight, duration of labor, gestational age, presence of meconium, professional cadre, and availability of priority equipment for NR were associated with the outcome of interest. But, after adjusting in the multivariable analysis, duration of labor, gestational age and availability of priority equipment for NR was found to have significant statistical association with neonate’s survival at time of discharge**..**

**Table 5:** Association between explanatory variables and survival of neonates after undergoing neonatal resuscitation in Ethiopia, EmONC Survey, 2016 (*N* = 555)

Characteristics	Survival of neonate’s at time of discharge, n (%)		OR(95%CI)	
	Survive	Not survive	Crude	Adjusted
Professional Cadre				
MD/HO	12 (75.0)	4 (25.0)	0.39 (0.081.80)	
Midwives	420 (83.3)	84 (16.7)	0.65(.22–1.88)	
Nurses	31 (88.6)	4 (11.4)		NS
Provider work experience in years				
< 2	202 (83.1)	41 (16.9)	0.75 (0.35–1.57)	
2–5	195 (82.6)	41 (17.4)	0.72(.34–1.52)	
> 5	66 (86.8)	10 (13.2)		NS
Age of provider in completed years				
≤ 25	272 (81.4)	62 (18.6)	1.1 (0.30–4.0)	
25–40	179 (86.9)	27 (13.1)	1.66(.44–6.26)	
> 40	12 (80.0)	3 (20.0)		NS
Sex of care provider				
Female	287 (84.2)	54 (15.8)	1.15(.73–1.81)	
Male	176 (82.2)	38 (17.8)		NS
Type of resuscitation				
Stimulation	7 (77.8)	2 (22.2)	0.35 (0.26–4.65)	
Bag and mask	306 (84.1)	58 (15.9)	0.53 (0.07–4.20)	
Stimulation with bag & mask	140 (81.9)	31 (18.1)	0.45 (0.06–3.66)	
Intubation (Ref)	10 (90.9)	1 (9.1)		NS
Mode of delivery				
Spontaneous Vaginal	420 (83.2)	85 (16.8)	1.45 (0.52–4.05)	
Instrumental	26 (92.9)	2 (7.1)	3.82 (0.66–22.0)	
Caesarian section (Ref)	17 (77.3)	5 (22.7)		NS
Birth weight (gram)				
< 2500	82 (77.4)	24 (22.6)	0.61 (0.36–1.03)	
≥ 2500 (Ref)	381 (84.9)	68 (15.1)		NS
Duration of labor				
≤ 12 h	161 (89.9)	18 (10.1)	2.2 (1.26–3.81)	**1.76 (1.23–3.13)**
> 12 h (Ref)	302 (80.3)	74 (19.7)		
Gestational age (weeks)				
< 37	47 (79.7)	12 (20.3)	1.3 (0.60–2.63)	**1.37 (0.61–3.10)**
≥ 37	304 (87.4)	44 (12.6)	2.2 (1.36–3.63)	**1.82 (1.09–3.04)**
Unknown (Ref)	112 (75.7)	36 (24.3)		
Mother/baby referred from another facility				
Yes	24 (82.8)	5 (17.2)	1.1 (0.39–2.83)	
No (Ref)	439 (83.5)	87 (16.5)		NS
Meconium present				
Yes	85 (90.4)	9 (9.6)	0.48 (0.23–0.98)	
No (Ref)	378 (82.0)	83 (18.0)		NS
Facility has separate newborn corner room				
Yes	197 (84.9)	35 (15.1)	1.21(.76–1.91)	
No (Ref)	266 (82.4)	57 (17.6)		NS
Facility has separate NICU room				
Yes	69 (83.1)	14 (16.9)	1.03(.55–1.91)	
No (Ref)	394 (83.5)	78 (16.5)		NS
Facility has staff rotation policy for newborn care				
Yes	207 (83.1)	42 (16.9)	0.96 (0.61–1.51)	
No (Ref)	256 (83.7)	50 (16.3)		NS
Facility type				
Hospitals	91 (85.0)	16 (15.0)	1.2 (0.65–2.09)	
Health centers (Ref)	372 (83.0)	76 (17.0)		NS
Facility location				
Urban	238 (84.1)	45 (15.9)	1.1 (0.71–1.73)	
Rural (Ref)	225 (82.7)	47 (17.3)		NS
Operating agency				
Government	446 (83.5)	88 (16.5)	1.19 (0.39–3.63)	
Private (Ref)	17 (81.0)	4 (19.0)		NS
Facility has care providers trained on NR				
Yes	393 (83.4)	78 (16.6)	1.01 (0.54–1.88)	
No (Ref)	70 (83.3)	14 (16.7)		NS
Availability of essential medicine and commodities	463 (83.4)	92 (16.6)	0.96 (0.81–1.14)	NS
Availability of priority equipment’s	463 (83.4)	92 (16.6)	0.79 (0.64–0.98)	**1.24 (1.09–1.54)**
Infrastructure components	136 (85.5)	23 (14.5)	1.1 (0.82–1.50)	NS
Availability of neonatal resuscitation guideline				
Yes	384 (83.8)	74 (16.2)	1.2 (0.67–2.09)	
No (Ref)	79 (81.4)	18 (18.6)		NS

Ref*: Reference category

NS*: Not statistically significant variable

Resuscitated newborns delivered below and 12 h of labor were 1.76 times (AOR = 1.76; 95% CI (1.23, 3.13) more likely to survive than those delivered after duration of greater than 12 h.

In addition, the neonate’s gestational age is significantly associated with the neonate’s survival status after resuscitation. As the gestational age increase, the chances of getting survive would also increase. Resuscitated newborns delivered at gestational age of greater than 37 weeks and above had 1.82 times increased chances of survival when compared to newborns with unknown gestational age (AOR = 1.82; 95% CI (1.09–3.04).

Facilities with one unit increase in the availability of priority equipment increases by 1.24 times the survival of the neonate after neonatal resuscitation performed (AOR = 1.24; 95% CI: 1.09, 1.54; *p* = 0.05).

## Discussion

More than two-third of the resuscitated babies were survived after NR in health facilities of Ethiopia and gestational age, priority equipment for NR and duration of labor were independently associated factors of survival of resuscitated neonates. Thus, this finding has implications both at the health facility level and the HCP level for the fight against neonatal mortality due to birth asphyxia. Appropriate caring for premature newborns and use of partograph to monitor each woman continuously throughout the duration of labor is very important intervention in low-resource settings as prolonged labor and delay in decision making are important causes of adverse obstetric outcomes. Besides, health facilities should invest more in ensuring that the availability of priority equipment’s for NR to perfectly perform the procedure within the golden minute [16].

Our study showed that, availability of priority equipment in facilities increases the survival of neonates after neonatal resuscitation. One unit increase in the availability of priority equipment’s in a health facility survival of the neonate increased by 1.24 unit (95% CI: 0.99, 1.54; *p* = 0.05). This implies the benefit of the preparation of essential equipment, and sometimes staff for unforeseeable and foreseeable resuscitations helps them to start ventilation on time, and increases the chances of a baby surviving after resuscitation. By improving the availability and readiness of NR equipment, Ethiopia can reduce barriers to the proper neonatal resuscitation practice and improve performance that impact to decrease high neonatal mortality in the country [17]. This is supported by evidence from an effective intervention to decrease global neonatal mortality; effective NR could prevent neonatal deaths by 30% as well as improve the outcomes of newborns delivered with birth asphyxia [18]. However, to ensure that newborns benefit from NR, essential equipment, medical supplies, need to be made available and ready at the right place for the right application of the procedure [19]. When health facilities are performing low on the availability and readiness measures for NR service, they cannot meet the practice and quality standards [20] and the potential health benefits of NR practice to newborns becomes compromised.

Prematurity is among the top three causes of neonatal mortality in Ethiopia [21] and the leading cause globally [6]. Resuscitated newborns with gestational age ≥ 37 weeks had 1.82 times increased chances of survival in our findings. This is in line with the study conducted in Tanzania [22] which shows newborns who died as compared to those who survived had significantly associated with gestational age and in Kenya, study indicated that gestation age ≥ 37 weeks were significantly associated with increased survival at 1 h post NR (OR = 1.38, *p* = 0.007, CI = 1.10–1.75). This can be explained by the fact that a preterm baby who is failing to establish regular respiration needs more swift support and those babies who are extremely bruised at delivery during resuscitation generally have an extremely poor outcome [23]. Again preterm babies, possibly through its well described negative effects on respiratory function, i.e. Inhibiting the action of surfactants and increased risk of sepsis [24].

The other predictor variable that affects the neonatal outcome after NR was the duration of labor. Resuscitated newborns delivered 12 h and less duration of labor was 1.76 times more likely to survive than those delivered greater than 12 h (AOR = 1.76; 95% CI (0.99, 3.13)). This might be because of prolonged duration of labor puts children at risk for developing brain damage that leads to cerebral palsy as a result of prolonged oxygen deprivation to the fetus or newborn, and the longer the baby is deprived of oxygen, the more severe the damage may be and get die [25].

We found that health care providers trained in NR do not have any significance on the outcome of a newborn after NR. This is because many healthcare providers had received training on NR either internally or from the district and national level, but many times the training was not provided when the health workers are offering newborn care (hands-on), which might have contributed to inadequate skills in newborn resuscitation. Another possible explanation for insufficient skill could be allocation of staff as described by Vesel et’al in Ghana [26]. An additional possible explanation could be the low proportion of non-trained providers that limits the statistical effect in this study.

Besides the above, although secretion obstruct babies airway and worsening asphyxia, our findings showed that newborns born with meconium (stained amniotic fluid in the airway who did not start breathing on their own) had no effect on chances of survival after neonatal resuscitation. However, this contradicts with other study done in Kenya [27, 28] which demonstrated that meconium presence was a predictor of survival of neonates after neonatal resuscitation. The possible justification could be, competency of health workers were good in airway clearance in presence of meconium in babies who did not start breathing on their own. As a result, many newborns born with meconium may not suffer both short term and long term squeal ranging from birth asphyxia and eventual early neonatal death in our study.

## Limitation of the study

This study had limitations. First, our measure of NR process quality was limited. We simply extracted from asphyxiated babies charts whether or the neonates were survived or not. Second, this study did not consider possible maternal factors that could have led to the deaths of the resuscitated newborns. Thus, better measurement methods such as direct observations would provide a better assessment of quality. The EmONC survey only reviewed the most three recent charts of babies who had faced with the difficulty of breathing delivered in the past 12 months. Therefore, many facilities did not have any charts and were not included in our analysis of NR process quality and outcomes. However, a significant strength of this study is that it used data from the EmONC survey which is a census of all health facilities, both public and private, that provided maternal and newborn health services in Ethiopia. Our study also used a combination of methods to assess quality including structural quality based on facility questionnaires and process quality based on chart extractions.

## Conclusions

Overall, the availability of priority equipment for NR service as a structural quality indicator is low in Ethiopia. However, more than two-third of the resuscitated babies were survived at the time of discharge. Regarding the predictors; gestational age, priority equipment for NR and duration of labor were the main correlates of neonatal outcome after NR service received.

Thus, we recommend that, efforts to avail of the priority equipment’s and supplies noted to be unavailable including appropriately mucus trap for suction and radiant warmer for each unit will ensure that the health workers are adequately equipped during deliveries to overcome immediate critical gaps and more attentive care and strong follow up should be given for premature and for those neonates had more than 12 h duration of labor to improve their quality of life.

Further observational research also warranted to measure quality of NR and its effect on resuscitated newborns.

## Data Availability

The datasets generated and/or analyzed during the current study are not publicly available due to they were collected for other purpose but are available from the corresponding author on reasonable request.

## Abbreviations

EDHS: Ethiopian demographic health survey
EmONC: Emergency obstetrics and newborn care
HCP: Health Care providers
NR: Neonatal resuscitation
